# Women’s Age at Marriage and Obesity in Pakistan

**DOI:** 10.1016/j.ehb.2025.101525

**Published:** 2025-08-23

**Authors:** Wiktoria Tafesse, Akseer Hussain, Marc Suhrcke

**Affiliations:** a Centre for Health Economics, University of York, United Kingdom; b Luxembourg Institute of Socio-Economic Research (LISER), Luxembourg

**Keywords:** Obesity, Female marital age, Pakistan

## Abstract

Obesity is a growing public health concern, particularly in low- and middle-income countries, with women facing a disproportionate burden. In Pakistan, the prevalence of female obesity is among the highest in South Asia. Although socio-economic and demographic factors contributing to obesity have been extensively studied, less is known about the role of age at marriage as a potential determinant of female obesity. This study examines the relationship between age at marriage and the risk of obesity in Pakistani women using data from the 2012-13 and 2017-18 Pakistan Demographic and Health Surveys. Baseline OLS models show that a higher age at marriage is significantly associated with a reduced risk of obesity, with stronger effects observed among younger cohorts. Instrumental-variable estimates, using community norms around female marital age, confirm the OLS results for urban women, indicating that each additional year of delayed marriage lowers the risk of obesity by 0.7 percentage points. Analysis of potential mechanisms identifies fertility patterns, schooling, literacy, labour-force participation, health knowledge and a reduced spousal age gap as key pathways linking early marriage to higher obesity risk among urban women.

## 1 Introduction

Obesity is rapidly increasing across the world, with low- and middle-income countries (LMICs) experiencing the highest rise. In South Asia, the increasing prevalence of obesity contributes to a double burden of malnutrition, as it coexists with high rates of under-nutrition (Popkin et al., 2020), which poses significant public health challenges and drives increased healthcare costs (Okunogbe et al., 2021). High body weight is a critical risk factor for multiple non-communicable diseases (NCDs), and this risk is particularly acute among South Asians (Sharma et al., 2017; Bishwajit, 2015). As a result, addressing obesity has become a pressing policy issue across several LMICs in the region.

Pakistan, with the highest obesity rate in South Asia after the Maldives, exemplifies this challenge (Awasthi et al., 2023). Nationwide data from 2017-2018 shows that over 38% of the adult population is overweight or obese, and the prevalence among women is even higher, at 52% (Awasthi et al., 2023). This gender disparity in obesity is not unique to Pakistan (Ameye and Swinnen, 2019), but may be heightened by deeply entrenched gender inequality and limited female empowerment, which are among the lowest globally (Alvarez-Saavedra et al., 2023; Awasthi et al., 2023; UNICEF, 2023). Existing research has documented a range of socio-economic and demographic correlates of obesity, such as urban residence, higher household wealth, and older age (Janjua et al., 2015; Alam et al., 2024). However, less attention has been given to gender-specific social norms and practices that may influence women’s health outcomes.

Age at (first) marriage is a key determinant of female empowerment, and has been linked to various adverse health outcomes, including mental health issues, poor reproductive outcomes, and reduced human capital (Fan and Koski, 2022). Although some studies have explored the relationship between early marriage and obesity in other South Asian countries and parts of Africa, the evidence base is correlational and mixed (Fan and Koski, 2022; Efevbera et al., 2019; Datta and Fazlul, 2023; Yusuf et al., 2018; Alvarez-Saavedra et al., 2023). Some research indicates that women who marry early are at greater risk of becoming obese, while others suggest a higher likelihood of being underweight (Yusuf et al., 2018; Efevbera et al., 2019; Datta and Fazlul, 2023; Alvarez-Saavedra et al., 2023). There are many potential suggested mechanisms - depending on the stage of the nutrition transition - via which early female marriage may impact body weight in LMICs: adverse fertility outcomes, human capital accumulation, decision making power, women’s mental health and psycho-social wellbeing (Efevbera et al., 2019; Datta et al., 2022a; Datta and Fazlul, 2023; Aiyar et al., 2021a). However, the association between age at marriage and health outcomes, including body weight, is prone to endogeneity. Women from poorer natal households are more likely to marry at younger ages, and unobserved factors may influence both marital timing and BMI, particularly when using cross-sectional data. Additionally, although less likely in the context of Pakistan’s arranged marriage system, reverse causality cannot be entirely ruled out if pre-marital health or body weight affects marriage prospects.

In this paper, we add to the growing body of literature by examining the causal relationship between age at marriage and the risk of obesity among women in Pakistan. We use data from the 2012-13 and 2017-18 Pakistan Demographic and Health Survey (PDHS) to assess whether marrying at a younger age increases the likelihood of obesity. OLS regressions show that delaying marriage by one year reduces the risk for obesity later in life by 0.5 percentage points, on average, for the pooled sample. Thus our baseline findings are similar to previous studies which observe that child marriage is associated with obesity risk in India and Tajikistan (Singh et al., 2025; Datta and Haider, 2022; Datta and Fazlul, 2023; Datta et al., 2023a). Moreover, we show that marrying at age 23, and above, has a particularly protective effect, compared to marrying below age 17, adding a more nuanced perspective compared to earlier studies, which exclusively focused on child marriage (Efevbera et al., 2019; Datta and Haider, 2022; Datta and Fazlul, 2023; Alvarez-Saavedra et al., 2023; Datta et al., 2023a).

While we observe a negative relationship across all age groups, the strongest protective effect of delayed marriage on later obesity is found for women aged 25-34 at the time of the survey. Similarly, the largest effect is observed for the youngest cohorts, highlighting the important role of economic development and changing nutrition transition as previously reported in related studies using data from Tajikistan and China (Datta et al., 2023a; Quan and Zhang, 2024). Despite the obesity prevalence being double that of the wealthiest women, compared to the poorest women in our sample, we find similar effects for women in the poorest household wealth quintile and in the two richest wealth quintiles. Our findings are in line with a study on India where child marriage was found to impact obesity across all wealth groups (Datta and Fazlul, 2023).

Our results highlight an important heterogeneity in that the effect is driven by women residing in urban areas, for whom older age at marriage decreases the likelihood of living with obesity by 0.6 percentage points. Given the potentially endogenous nature of marital age in relation to human capital, we follow previous literature on child marriage in Pakistan by Mughal et al. (2023) and Ashok et al. (2024), and instrument for marital age with prevailing community-norms for marital age by community. Our IV results confirm the OLS results for the urban sample, by showing that delaying marital age by one year reduces the risk of obesity by 0.7 percentage points. Focusing on the urban sample and relying on our IV framework to inspect potential mechanisms, we find that marrying later improves a number of fertility outcomes as observed in the previous literature for LMICs (Yaya et al., 2019; Chari et al., 2017; Fan and Koski, 2022; Efobi, 2024). Older marital age reduces premature child bearing, the number of children and the frequency of short-spaced pregnancies, which are all factors impacting long-term weight retention and obesity risk (Gunderson et al., 2000; Hanson et al., 2015; We et al., 2016; Patchen et al., 2017; Davis et al., 2014; Hutcheon et al., 2019; Mannan et al., 2013). Delaying marriage also improves schooling, literacy, labour force participation and general health knowledge which may in turn reduce the risk for obesity through better nutrition investment and health behaviours as suggested by Jensen and Thornton (2003); Field and Ambrus (2008); Chari et al. (2017); Asadullah and Wahhaj (2019). Additionally, older marital age reduces the spousal age-gap which is closely associated with female decision-making power, which in turn had been shown to impact household dietary decisions (Alvarez-Saavedra et al., 2023; Chari et al., 2017).

Thus, we show that early marriage can act as a catalyst for obesity among urban women in countries undergoing a nutrition transition, by limiting education, health knowledge, fertility control and bargaining power. Our findings contribute to the literature on the determinants of obesity in Pakistan and emerging economies more broadly, highlighting how gendered social norms and urbanisation shape rising obesity risks (Janjua et al., 2015; Ferdausi et al., 2022; Waghmare et al., 2022a; Abay and Amare, 2018; Aiyar et al., 2021a,b; Ameye and Swinnen, 2019; Costa-Font and Györi, 2020).

## 2 Literature review and conceptual framework

### 2.1 Literature review

There is a growing body of literature examining the relationship between child marriage and poor nutritional outcomes across developing countries. Most studies rely on correlational analyses of cross-sectional Demographic and Health Surveys (DHS). Goli et al. (2015); Das and Adhikari (2021); Mim et al. (2024); Yusuf et al. (2018) report that child marriage is associated with a higher risk of underweight in India, Bangladesh, and Nigeria. Conversely, Efevbera et al. (2019) find a reduced risk of underweight among women who were married as children compared to those married as adults, using pooled DHS data from multiple Sub-Saharan African countries. Datta et al. (2023a) observe that women who married before the age of 18 face an elevated risk of obesity in adulthood in Tajikistan; their quantile regression results show that child brides have a higher BMI than their peers across the entire BMI distribution. Similarly, Datta and Fazlul (2023) report that women married in childhood are at greater risk of overweight and obesity in India. Accounting for household wealth, child marriage is further associated with increased risks of both overweight and anaemia in India (Datta and Haider, 2022). Moreover, several studies demonstrate that child marriage is linked to a higher risk of obesity-related conditions such as hypertension and elevated blood glucose among women in India (Datta and Tiwari, 2023; Tiwari et al., 2023; Datta et al., 2023b, 2022b).

Alvarez-Saavedra et al. (2023) analyse two rounds of panel data for India, employing fixed effects methods and find that child marriage increases the risk of underweight. Although this study represents a methodological improvement over the existing literature, panel data techniques cannot fully eliminate concerns about endogeneity, particularly those related to selection bias. Furthermore, similar to previous studies Datta et al. (2023a); Datta and Fazlul (2023); Datta and Haider (2022), Alvarez-Saavedra et al. (2023) control for variables that may themselves be affected by child marriage, raising concerns about post-treatment bias.

A related strand of the literature investigates the impact of marital transitions on BMI. Analysing longitudinal data from China, Tang et al. (2024) find that marriage increases the probability of obesity by 2.8%, with stronger effects observed among those who married at younger ages compared to older individuals. Another panel data study from China by Quan and Zhang (2024) reports that men’s BMI increases during the first five years after marriage and then stabilises, while no clear trend is observed for women. Moreover, the authors find an upward trend in the average treatment effects of marriage on male BMI over time. Similarly, Datta et al. (2023a) observe that the effect of child marriage on female obesity in Tajikistan is more pronounced among younger cohorts.

These findings suggest that marriage, including early marriage, contributes to increased BMI during the later stages of the nutrition transition, when economic development leads to obesogenic environments through changes in food systems, the increased availability of high-calorie foods, and more sedentary lifestyles (Swinburn et al., 1999). Evidence from high-income settings supports this interpretation, as most studies report a positive association between marital status and higher body weight (Sobal et al., 1992, 2003; Averett et al., 2008; Teachman, 2016; Averett et al., 2013; Malcolm and Kaya, 2016; Sato, 2021; Dinour et al., 2012; Nikolic Turnic et al., 2024; Tang et al., 2024; Quan and Zhang, 2024). Pakistan is currently in the early to intermediate stages of the nutrition transition, where high rates of both underweight and overweight coexist. Urban areas are at more advanced stages of the nutrition transition, characterised by shifts in the food environment, increased consumption of energy-dense processed foods, more sedentary occupations and leisure activities, and greater reliance on motorised transport (Popkin, 1998; Aiyar et al., 2021a). These transitions are reflected in substantially higher levels of obesity in urban compared to rural areas of Pakistan (Janjua et al., 2015; Alam et al., 2024). Consequently, the relationship between early marriage and obesity risk among women in Pakistan is likely to differ between urban and rural women.

### 2.2 Potential mechanisms

The relationship between age at marriage and adult body weight may, in part, reflect selection effects. Girls who marry at younger ages are more likely to come from poorer households and to be underweight before marriage (Jensen and Thornton, 2003; Rasul et al., 2022; Datta and Haider, 2022). The impact of this pre-marital selection on later-life body weight depends on the wealth status of the marital household, the food environment, and the broader relationship between wealth and BMI. If a woman remains in a food-insecure environment after marriage, childhood undernutrition may increase the likelihood of remaining underweight in adulthood. Conversely, Efevbera et al. (2019) hypothesise that girls who marry as children may gain access to more abundant food, thereby reducing their risk of underweight in later life. Moreover, childhood stunting may predispose individuals to obesity when later exposed to obesogenic environments during adolescence and early adulthood (Black et al., 2013a).^1^

Beyond selection effects, several mechanisms may explain how early marriage itself, independent of the poverty often associated with it, can place women on a trajectory towards overweight and obesity later in life. Below, we outline key pathways identified in the existing literature that are particularly relevant to understanding the link between early marriage and female obesity in the context of Pakistan.

First, early marriage may influence body weight through its effects on human capital accumulation. At a theoretical level, early marriage is hypothesised to interrupt investments in education and skill accumulation leading to lower earnings, health knowledge and worse long-term health outcomes (Becker, 1962, 1965, 1985, 2007). Lower education is also suggested to reduce the efficiency of utilising health inputs to improve health by impacting individuals’ capacity to access, process and evaluate health-related information (Grossman, 1972, 2003; Cutler and Lleras-Muney, 2010) These predictions are supported by empirical results across LMICs as child marriage has been shown to reduce girls’ education, resulting in lower literacy, reduced access to information, and diminished engagement with health-promoting behaviours and investments (Jensen and Thornton, 2003; Field and Ambrus, 2008; Chari et al., 2017; Asadullah and Wahhaj, 2019). Another pathway may stem from decreased female labour force participation which has been linked to higher BMI in India and Pakistan, explained by reduced caloric expenditure (Dang et al., 2019; Janjua et al., 2015). Furthermore, lower female earnings limit women’s control over productive household assets, including decisions about nutrition and health investments (Subramanee et al., 2022).

Second, fertility may serve as a mechanism linking early marriage to later-life obesity. Marrying in childhood or at a young age increases the likelihood of early childbearing, higher total lifetime fertility and shorter birth intervals between pregnancies (Yaya et al., 2019; Chari et al., 2017; Efevbera et al., 2019; Fan and Koski, 2022). Childcare demands on women’s time also reduce time for the production of health of the mothers (Becker, 1965; Grossman, 1972). Applying a life-course perspective, the transition into parenthood has been hypothesised to influence women’s weight gain through mechanisms such as increased psychosocial stress and changes to health-promoting behaviours, while an earlier timing of this transition is expected to generate cumulative and persistent health effects over time (Elder et al., 2003; Umberson et al., 2011). Empirical evidence shows that early childbearing, including a short interval between menarche and first birth, as well as adolescent pregnancies, is associated with greater gestational weight gain and a heightened risk of being overweight post-pregnancy (Gunderson et al., 2000; Hanson et al., 2015; Umberson et al., 2011; We et al., 2016; Patchen et al., 2017). A higher number of pregnancies contributes to incremental weight gain over time, while frequent short inter-pregnancy intervals (less than 12 months) are particularly associated with an increased risk of subsequent obesity (Davis et al., 2014; Hutcheon et al., 2019). Given consistent findings that women who gain excess weight during pregnancy often do not return to their pre-pregnancy weight, such fertility patterns may have cumulative and long-term implications for female obesity (Mannan et al., 2013).

However, in food-insecure settings, adverse fertility experiences — including adolescent pregnancies and closely spaced births, have also been linked to lower BMI (Nigatu et al., 2018; Wells et al., 2022; Adair, 2004; Aiyar et al., 2021a). The relationship between fertility and nutritional outcomes likely depends on the stage of the nutrition transition. Aiyar et al. (2021a) develop an extended framework of the nutrition transition model by Popkin (1998); Swinburn et al. (2019) to explain the emergence of within-country differences in overweight patterns in India and propose that lowering of reproductive stress is correlated with higher overweight rates but depends on the level of development. For example, Were et al. (2020) find that parity is associated with underweight in sub-Saharan African countries with high under-nutrition rates, but is a risk factor for overweight and obesity in countries where excess weight is more prevalent. On the other hand, research on Pakistan reports that previously malnourished women gained more weight than their marginally nourished counterparts after experiencing high levels of reproductive stress (Winkvist et al., 1994).

Third, the effect of early marriage on female empowerment may also contribute to later-life obesity risk (Alvarez-Saavedra et al., 2023). Women who marry during adolescence have been shown to experience reduced decision-making autonomy, bargaining power, and mobility (Jensen and Thornton, 2003; Field and Ambrus, 2008; Efobi, 2024). A substantial body of literature highlights the critical role of intra-household bargaining power in shaping household resource allocation and nutrition outcomes (Richards et al., 2013). Where women possess greater decision-making power and influence over resource distribution, household spending on health and education is typically higher (Santoso et al., 2019), which may in turn support healthier behaviours and improved nutritional outcomes. Chari et al. (2017) and Efobi (2024) also find that child marriage has intergenerational effects on children’s nutritional status in India and Ethiopia, respectively, with reduced female autonomy identified as a key explanatory factor.

Fourth, marrying at a young age may also have adverse psychosocial consequences (Efevbera et al., 2019; Burgess et al., 2022; Datta et al., 2023a), including an increased risk of mental ill-health and exposure to domestic violence (Roychowdhury and Dhamija, 2021). These experiences may have long-term implications for body weight, given the well-established association between depression and obesity (Avila et al., 2015). Exposure to domestic violence, in particular, has been linked to both obesity and underweight in women, highlighting the complex and context-dependent nature of this relationship (Yount and Li, 2011; Ackerson and Subramanian, 2008).

## 3 Data

We analyse individual-level data from the 2012-13 and 2017-18 PDHS, which has the advantage of containing women’s objectively measured weights and heights. The PDHS applies a stratified two-stage survey sampling design to provide nationally, as well by rural and urban level, representative samples of women of reproductive age (i.e., 15 to 49 years old). The survey collects detailed health and socio-economic information on eligible women and their households. We limit the sample to women 18-49 years of age, with available information on weight and height, who are neither pregnant nor breastfeeding at the time of the survey, to circumvent confounding effects from childhood and most recent childbirth on body weight. From 10,001 individual observations with available weight and height information, we are left with 6,153 observations in our final analytical sample.

## 4 Empirical strategy

We estimate the following Ordinary Least Squares (OLS) model to assess the relationship between age at marriage and the probability of living with obesity.

(1) $${\text{Obese}}_{icdt}=\alpha_{0}+\beta Marriageage_{it}+\delta X_{it}+\lambda CommSESS_{c}+\phi_{d}+\sigma_{t}+\mu_{icdt}$$

*Obese*_*icdt*_ is a binary outcome variable representing the risk of obesity for woman *i*, in community *c* and district *d* at survey year *t*. We use the Asian-specific BMI threshold recommended by the WHO of BMI ≥25 as our outcome measure of adiposity (Pigeyre et al., 2018). In addition we conduct sensitivity checks using continuous BMI and the Asian specific definitions for overweight (BMI ≥ 23.0) and underweight (BMI < 18.5).

*Marriageage*_*it*_ denotes a woman’s continuous age at first marriage. The key determinant of interest is a woman’s retrospectively reported age at first marriage, including cohabitation. This continuous variable is calculated in the data by asking the woman about her birth date and the date she began living with her first spouse or partner. This age is taken as including up to the next completed year of age and is truncated to full years. In addition, we construct a categorical variable for age at first marriage quintiles to investigate non-linear associations.

We control for plausibly exogenous individual-, household- and community level co-variates which may impact age at marriage and obesity in adulthood.

*X*_*it*_ is a set of individual and household covariates. As countries develop and reach later stages of the nutrition transition, age becomes strongly associated with overweight (Aiyar et al., 2021a) which motivates accounting for respondents’ current age. Given that early marriage, and child marriage in particular, is associated with prior individual childhood factors such as poor economic background (Subramanee et al., 2022), it is important to partial out pre-marital socio-economic status. However, as such information is not available in the PDHS, we proxy for poor childhood socio-economic status by including a binary variable denoting adult short stature. Short stature is correlated with stunting in early life and childhood stunting has been shown to be a predictor of metabolic risk factors in adulthood (Hoddinott et al., 2013). While genetics determine the growth potential of an individual, poverty, impacting the nutrition and disease environment in early life, is a well established cause of stunting (Black et al., 2013b; Lui et al., 2024). We define short stature for an adult woman as being below 150 cm based on the WHO definition (WHO Multicentre Growth Reference Study Group., 2006). Studies using data for Pakistan and other emerging economies confirm the commonly observed relationship between correlates of obesity across LMICs, such as urbanicity and community socio-economic status which is likely to reflect varying degrees of economic development and thus the obseogenic environment (Popkin et al., 1995; Jones-Smith and Popkin, 2010; Aiyar et al., 2021a,b; Janjua et al., 2015; Biswas et al., 2023; Waghmare et al., 2022b; Alam et al., 2024). Therefore we include a binary variable for urban compared to rural residence.

Efevbera et al. (2019) emphasise the importance of controlling for community-level variation due to local contextual factors that jointly determine marriage and nutritional outcomes. Therefore, we follow Janjua et al. (2015) in constructing a measure of the average community household wealth, i.e. the enumeration area in the PDHS, from the composite measure of a household’s asset ownership transformed into household wealth quintiles, denoted *CommSES*_*c*_.

*ϕ*_*d*_ are district fixed effects which capture all unobserved time-invariant factors across districts. *σt* are the survey-wave fixed effects, accounting for time effects including nationwide changes in the food environment and *µ*_*icdt*_ is the standard error clustered at the district level. We present our main regression results for the pooled sample as well as for rural and urban samples separately given the variation in marriage practices and the obesogenic environment across rural and urban areas in Pakistan.

### 4.1 Descriptive Statistics

The descriptive statistics for the pooled, rural and urban analytical samples are shown in Table 1.

We note that the average BMI for the pooled sample is 25.84 which corresponds to the obese category according to the Asian specific BMI-cut offs. 52% of the sample are classified as obese and 7% are underweight. The proportion of obese women is higher for the urban sample compared to the rural sample, where almost two-thirds of women are classified as obese in urban areas. The average age at marriage is 19.11 years for the pooled sample. Mean marital age is lower for rural areas (18.55 years) and higher for urban areas (19.67 years). Average current age is 35.38 for the pooled sample. One fifth of all women in our sample have short stature.

## 5 Results

### 5.1 Main Results

The regression results for the pooled (columns 1 - 3), rural (columns 4 - 6) and urban (columns 7 - 9) samples are presented in Table 2. The first specification for each sample shows the relationship between age at (first) marriage and obesity without any covariates. The second specification includes controls for district fixed effects, urban or rural residence, survey fixed effects, the woman’s current age and a dummy variable for short stature. Lastly, the average community wealth score is included to account for area-level socio-economic differences.

The bivariate regression points to a positive relationship between marital age and risk of living with obesity for the pooled and rural samples, see columns 1 and 4 in Table 2. For the urban sample, on the other hand, we do not observe a relationship between age at marriage and obesity, see column 7. Including covariates for location, survey wave, age and short stature, reverses the relationship and yields a negative and statistically significant coefficient of -0.003 for the pooled sample (column 2). Accounting for variation across community wealth increases the magnitude of the coefficient of interest to -0.005, see column 3. The reversal of the relationship between marital age and current obesity across bivariate and multivariate regressions is similar to the findings of Datta et al. (2023b); Datta and Haider (2022); Datta and Fazlul (2023); Datta et al. (2023a) for child marriage on the risk of overweight and obesity in India and Tajikistan. As hypothesised by Datta et al. (2023b); Datta and Haider (2022); Datta and Fazlul (2023), this may be explained by the selection effect reflecting that women who marry at a very young come from poor households with a higher risk of being underweight.

Accounting for the full set of covariates, marrying one year later reduces urban women’s risk of obesity by 0.6 percentage points, see column 9. Including covariates for the rural sample results in a positive but not significant negative effect, see column 6. Heterogeneous effects of gender norms on women’s body weight across urban and rural location are in line with Alvarez-Saavedra et al. (2023).

In addition, we observe that residing in an urban area is associated with a 15.3 percentage points higher likelihood of living with obesity. This positive association aligns with the nutrition transition framework and is consistent with previous evidence from Pakistan and other LMICs. (Janjua et al., 2015; Waghmare et al., 2022b; Popkin, 1998; Swinburn et al., 2019; Nie et al., 2019; Aiyar et al., 2021a; Abay and Amare, 2018; Aiyar et al., 2021b; Jones-Smith and Popkin, 2010).

Women surveyed in 2017–2018 compared to 2012–2013 have a higher risk of obesity which is in line with previously documented trends of rising obesity rates across South Asia and Sub-Saharan Africa (Abay and Amare, 2018; Aiyar et al., 2021a). Our findings also support earlier evidence showing that older age is associated with higher obesity risk among women in the Global South, including Pakistan (Aiyar et al., 2021a; Averett et al., 2014; Waghmare et al., 2022b; Abay and Amare, 2018). Being one year older increases the risk for obesity by 1 percentage point for the pooled sample. We also note a slightly stronger age effect for the urban compared to the rural sample.

Short stature is positively associated with obesity across all specifications and sub-samples but a statistically significant estimate is only found after accounting for community wealth for the pooled sample. This positive association is consistent with findings from countries undergoing a nutrition transition, and has been attributed to metabolic changes related to childhood stunting, which may increase the likelihood of excess weight gain when later exposed to high-fat diets (Popkin et al., 1996; Sawaya et al., 1998; Hoffman et al., 2000; Muhammad, 2018; Escher et al., 2024).

We include a control for average community wealth in columns 3, 6, and 9. Since average community wealth is typically higher in urban than in rural areas, including this variable attenuates the obesity-enhancing effect of urban residence. Residing in a community with a higher average wealth quintile increases the likelihood of female obesity by 11.9 percentage points for the pooled sample. Moreover, the effect of community wealth appears to be larger in rural compared to urban areas. This positive association between community level socio-economic factors and obesity risk is consistent with previous findings in Pakistan and other emerging economies (Popkin et al., 1995; Jones-Smith and Popkin, 2010; Janjua et al., 2015; Aiyar et al., 2021a,b).

We investigate non-linear effects by using a categorical variable for age at marriage denoting the quintiles of marital age. The regression results, accounting for the full set of controls, are shown in Table 3. Compared to marrying below age 17, we observe marrying at age 23 or above reduces the risk of developing obesity by 4.3 percentage points. We observe differences in protective effects by marital age categories by rural and urban residence. For women in rural areas, marrying at ages 20-22, compared to below age 17 is is associated with a reduction of 4.1 percentage points. For urban women the obesity reducing relationship is driven by marrying at ages 23 and above. Marrying at 23 or later reduces urban women’s later risk of living with obesity by 5.9 percentage points.^2^

### 5.2 Heterogeneity

Given that a woman’s age is a significant predictor of obesity, we estimate Equation 1 separately for women aged 24 or younger, 25–34, and 35 and above at the time of interview. The regression results are presented in Table 4. Accounting for the full set of covariates, we find that delaying marriage by one year reduces the risk of obesity by 0.8 percentage points for women aged 25–34. We observe half this effect size for women aged 35 and above. Although the coefficient is largest in magnitude for the youngest age group (18–24), it is not statistically significant, potentially due to the smaller sample size relative to the older groups. The larger impact observed among women under 35 may suggest that weight gain is more pronounced shortly after marriage, consistent with Quan and Zhang (2024) who report that post-marriage BMI increases most significantly during the initial period following marriage among men in China. However, it is difficult to disentangle age effects from cohort effects in our setting, given the cross-sectional nature of the PDHS.

Nonetheless, subsample analyses by age confirm that our baseline results are not sensitive to selection effects related to age or to recall bias. As the DHS only includes ever-married women of reproductive age, analyses using the full sample may underestimate age at marriage for younger cohorts compared to older ones (Rasul et al., 2022). Given that the majority of women aged 25 and older in Pakistan have ever been married (Rasul et al., 2022), the subsample analyses of older age groups indicate that our main findings are not driven by this sample selection. Erroneous recall of age at marriage, particularly among older women, may pose another source of bias (Coughlin, 1990; Ashok et al., 2024). However, since we observe protective effects of delayed marriage for women under the age of 35, our conclusions are unlikely to be affected by recall bias.

We inspect changes in the relationship between marital age and obesity over time by conducting sub-sample analyses by birth cohort. The regression results for women born in 1962-1974, 1975-1984 and 1985-2000, respectively, are presented in Table 5. The obesity protective effect of marrying later appears to be driven by women born in 1975 and later. Delaying marriage by one year reduces the risk for obesity by 0.5 percentage points for women born in 1975-1984. The effect doubles in magnitude to 1 percentage point for women born in 1985-2000 suggesting that economic development modifies the relationship between marital age and obesity. Our findings are comparable to Datta et al. (2023a) who report a positive relationship between child marriage and obesity in the youngest but not for the oldest birth cohort of Tajik women and Quan and Zhang (2024) who observe an upward trend in the average treatment effect of marriage on male BMI over time in China between 1989 to 2015.

Like many other LMICs, Pakistan is characterised by a wealth gradient in obesity (Janjua et al., 2015; Waghmare et al., 2022a). In our analytical sample, 29.4% of women in the poorest household wealth quintile are obese, compared to 68.4% of women in the richest quintile. At the same time, the average age at marriage is 17.7 years for women in the poorest households and 20.7 years for those in the richest. Given the diverging patterns in both obesity and age at marriage by wealth, we present the regression results by household wealth quintiles in Table 6.

Although we observe negative coefficients across all wealth subgroups, there are notable differences in magnitude and statistical significance. Marrying at a young age is significantly associated with an increased risk of obesity among women from both the poorest and the richer and richest households. This effect is evident at both ends of the socioeconomic spectrum and is comparable to the findings of Datta and Fazlul (2023), who report that child marriage increases the risk of obesity across all household wealth quintiles in India. These results suggest that the mechanisms linking child marriage to weight gain, after controlling for average community wealth, may operate in both poor and affluent households, albeit through different pathways. On the one hand, among economically disadvantaged Pakistani women, those who married early exhibit higher obesity prevalence relative to their low-income peers who married later. For the poorest women, early marriage may reduce the risk of underweight, as suggested by Efevbera et al. (2019), and thereby increase the risk of obesity for those at the margin. The relatively younger average age at marriage among poorer women may also strengthen the observed linear relationship between marital age and obesity in this group. On the other hand, for women from higher-wealth households, where baseline obesity rates are already high, early marriage appears to compound this risk.

### 5.3 Robustness

We find a similar relationship between age at marriage and the risk of obesity when accounting for district-level trends and community (DHS sampling cluster) fixed effects, see Table A1 in the Appendix. More importantly, we observe a negative and statistically significant estimate for the rural subsample after including community fixed effects, which further highlights the modifying role of the socio-economic environment as noted by Efevbera et al. (2019).

Changing the outcome to being overweight (and obese), using the South Asianspecific BMI cut-off (BMI ≥ 23.0), does not alter our conclusions for the pooled sample (see Table A2 in the Appendix). However, subsample analyses by rural and urban residence reveal a different pattern than that observed for obesity alone. We find a negative and statistically significant relationship between age at marriage and the risk of being overweight in the rural sample, but not in the urban sample, after including all controls. The relationship between marital age and continuous BMI is consistently negative and statistically significant across all subsamples, after accounting for all covariates (see Table A3). The bivariate relationship between marital age and the risk of being underweight (BMI < 18.5) supports the previously discussed selection effect, where underweight women are more likely to marry at a younger age. However, this relationship disappears after controlling for covariates, as shown in Table A4 in the Appendix. Moreover, results from a logit regression model further confirm the robustness of our OLS findings, see Table A5 in the Appendix.

### 5.4 Instrumental Variable estimation

Our OLS results may be subject to endogeneity arising from selection bias and omitted variable bias - particularly due to missing data on women’s pre-marital characteristics and caloric intake and expenditure. To address this, we exploit exogenous variation in women’s age at marriage derived from prevailing community norms prior to each respondent’s marriage and employ an Instrumental Variable (IV) regression approach.

Our IV strategy draws inspiration from previous studies examining the effects of child marriage on son preference and intergenerational human capital outcomes in Pakistan (Mughal et al., 2023; Ashok et al., 2024). In many traditional settings, including Pakistan, early marriage reinforces social norms around gender and sexuality. Accordingly, the community-level prevalence of early marriage serves as a strong proxy for normative pressure, influencing individual age at marriage (Mughal et al., 2023; Ashok et al., 2024). We therefore construct a community-level instrument, defined as the average marital age within a sampling cluster five years prior to each respondent’s year of marriage. These community-level norms regarding marital timing are plausibly exogenous to women’s later obesity risk, particularly after controlling for the socio-economic status of the community.

The second-stage IV results, which account for the full set of covariates, are presented in Table 7. The Kleibergen-Paap rk Wald F statistic is 412.8 for the pooled sample, indicating that our instrument is not weak. The IV estimates suggest that delaying marriage by one year reduces the risk of obesity by 0.7 percentage points for urban women, closely aligning with the corresponding OLS estimate. However, the IV coefficient for the pooled sample is not statistically significant, which may reflect the absence of a negative effect in the rural sample. The opposite (although statistically insignificant) signs of the OLS and IV coefficients for the rural sample may indicate a greater degree of bias in the rural estimates compared to the urban sample, or may reflect differing Local Average Treatment Effects across subpopulations.^3^

### 5.5 Mechanisms

Next, we explore whether the suggested mechanisms related to human capital, fertility and female empowerment discussed in Section 2 can explain the protective effect of delayed marriage on later female obesity in urban Pakistan. We rely on our IV strategy given the risk of endogeneity using OLS, and follow Chari et al. (2017) by regressing the possible mechanisms available in the PDHS on the instrumented age at marriage.

We investigate the following mediators related to human capital and health knowledge: whether the woman resides in a non-poor household (defined as the household not being in the bottom two wealth quintiles), the number of completed years of schooling, the ability to read a full sentence, general health knowledge, and whether the woman works outside the household. General health knowledge is a binary variable taking the value 1 if a woman is aware of any modern family planning methods and has ever heard of tuberculosis and sexually transmitted diseases, and 0 otherwise. The IV estimates show that delaying marriage does not significantly affect the likelihood of residing in a poor household. However, postponing marriage by one year increases women’s completed years of schooling by nearly half a year, improves literacy by 2.6 percentage points, increases the chances of being employed by 0.9 percentage points and raises general health knowledge by 1.8 percentage points (see Table 8). Thus, marrying later may reduce the risk of obesity by enhancing women’s knowledge, access to information, increased earnings and energy expenditure due to employment, and and capacity to make better-informed health and nutritional investments and decisions, as suggested by Jensen and Thornton (2003); Field and Ambrus (2008); Chari et al. (2017); Asadullah and Wahhaj (2019).

We find consistent effects across various fertility outcomes, as shown in Table 9. Marrying at a later age has a small but negative effect on the likelihood of ever having given birth and delays age at first birth by 0.87 years. The effects on age at first birth, as well as the probability of giving birth by age 18, may in part reflect a mechanical delay in childbearing, as most women in Pakistan have children shortly after marriage. Furthermore, we observe negative associations with adolescent pregnancy, the total number of children ever born, and the frequency of births with short birth intervals among urban women who have had more than one child. These findings are consistent with the hypothesised pathways discussed in previous studies (Yaya et al., 2019; Chari et al., 2017; Efevbera et al., 2019; Fan and Koski, 2022; Datta et al., 2022a). Thus, early marriage influences adverse fertility outcomes, which in turn shape the cumulative risk of obesity across a woman’s life course, as demonstrated by a substantial body of literature (Gunderson et al., 2000; Hanson et al., 2015; We et al., 2016; Patchen et al., 2017; Davis et al., 2014; Hutcheon et al., 2019; Mannan et al., 2013).

We follow Alvarez-Saavedra et al. (2023) in measuring female empowerment by constructing variables related to restricted mobility, decision making power, exposure to domestic violence and age difference with husband (age of the husband minus age of the wife) as a larger spousal age gap is associated with lower female bargaining power (Carmichael, 2011).

Women’s mobility is constructed by summing the values of three binary variables that capture constraints on a woman’s ability to move outside the home: (1) whether obtaining permission to seek medical help for herself is considered a major problem; (2) whether not wanting to go alone for medical help is considered a major problem; and (3) whether the respondent agrees that wife-beating is justified if a woman goes out without informing her husband. We define restricted mobility as a score of two or more of this index. We also construct a binary variable indicating a lack of decision-making power, defined as a woman having no say in any of the following domains: how her own or her husband’s earnings are spent, decisions regarding her own healthcare, large household purchases or visits to family or relatives.

From Table 10, we find no significant effects on these direct measures of gender inequality, such as restricted mobility, decision-making power, or having ever experienced domestic violence. However, measures of female empowerment based on standard survey questions may not accurately reflect true agency or bargaining power (Chari et al., 2017). On the other hand, marrying at an older age reduces the observed age gap with the husband, which is likely to enhance women’s bargaining power, potentially influencing decisions related to food purchases, physical activity and other health-related behaviours.

## 6 Discussion and conclusion

This study examines the relationship between age at marriage and female obesity in Pakistan, using pooled data from the 2012-13 and 2017-18 Pakistan Demographic and Health Surveys (PDHS). Our findings reveal that delaying marriage by one year reduces the risk of obesity later in life among urban women by 0.6 percentage points. Instrumenting marital age with prevailing community norms supports the robustness of these estimates. By providing causal evidence, our study contributes to the emerging literature on early female marriage and obesity in low- and middle-income countries (LMICs), which to date has largely relied on correlational analysis (Datta and Haider, 2022; Datta and Fazlul, 2023; Alvarez-Saavedra et al., 2023; Datta et al., 2023a).

Multiple sensitivity checks indicate that our results are robust to the inclusion of DHS cluster fixed effects, controlling for district-level trends and changing the outcome variable to continuous BMI. Moreover, our findings are not driven by women’s age at the time of the survey or by sample selection effects resulting from the inclusion of ever-married women. We document a continuous relationship between age at marriage and obesity, and demonstrate that marrying after adolescence, in particular, has a pronounced obesity-reducing effect. This adds nuance to previous work, which has focused exclusively on child marriage (Efevbera et al., 2019; Datta and Haider, 2022; Datta and Fazlul, 2023; Alvarez-Saavedra et al., 2023; Datta et al., 2023a).

Furthermore, both the OLS and IV results indicate that delaying marriage has a protective effect against obesity among women in urban Pakistan, where obesity prevalence is higher. Urban centres in Pakistan are further along in the nutrition transition, characterised by calorie-dense diets and reduced physical activity, making early marriage more likely to contribute to obesity risk in such environments. We do not find a consistent effect for women in rural areas, where food insecurity, physically demanding agricultural labour and underweight are more prevalent (Ferdausi et al., 2022). This heterogeneity aligns with the notion that the consequences of early female marriage for body weight depend on the degree of obesogenic exposure, and it complements the broader literature demonstrating that child marriage is associated with both obesity and underweight, contingent on local conditions (e.g. Yusuf et al. (2018); Efevbera et al. (2019); Alvarez-Saavedra et al. (2023); Datta and Fazlul (2023)).

Sub-sample analyses by birth cohort also suggest that economic development influences the relationship between early female marriage and obesity risk. For the youngest cohort of women, born between 1985 and 2000, each additional year of delaying marriage reduces the likelihood of living with obesity by one percentage point. The corresponding estimate is approximately halved for women born between 1975 and 1984 and we find no significant effects for the oldest cohort (1962–1974). Our cohort-based findings are consistent with Datta et al. (2023a), who report stronger effects of child marriage on female obesity among younger cohorts in Tajikistan, and with Quan and Zhang (2024), who find that the positive impact of marriage on BMI is more pronounced across time in China. Additionally, sub-sample analyses by household wealth reveal that the relationship between marital age and obesity risk is evident at both ends of the wealth spectrum, echoing previous findings on child marriage and obesity in India by Datta and Fazlul (2023).

Focusing on the urban sample and using our IV strategy, we examine the hypothesised mechanisms through which marital age influences female obesity, as suggested by previous literature. We find that delaying marriage increases years of education, literacy, probability of employment and general health knowledge — factors that are well-established as protective against obesity (Cutler and Lleras-Muney, 2010). Marrying at a later age also reduces the risk of several adverse fertility outcomes, including early childbearing, a higher number of children and more pregnancies with short birth intervals — all of which have been shown to contribute to obesity risk through weight gain and weight retention across the life course. These findings align with the mechanisms proposed by Datta et al. (2023a); Datta and Fazlul (2023). Furthermore, even though we do not find impacts on direct self-reported measures of female mobility and decision-making power, older age at marriage reduces the age gap between spouses, which has been associated with increased female bargaining power. This may, in turn, improve nutritional investments within the household (Jensen and Thornton, 2003; Santoso et al., 2019).

We demonstrate that early marriage can act as a catalyst for obesity among women in urban settings, primarily through its detrimental effects on female education, probability of employment, health knowledge, fertility outcomes, and spousal bargaining power. These insights add to the existing literature on the determinants of obesity in Pakistan (Janjua et al., 2015; Ferdausi et al., 2022; Waghmare et al., 2022a). Given the ongoing structural transformation and the rapid rise in obesity rates in rural areas of South Asia (Aiyar et al., 2021b), the influence of early marriage on obesity may become increasingly pronounced beyond urban contexts. Our findings thus contribute to the broader literature on the gendered determinants of obesity in emerging economies and their interaction with urbanisation (Abay and Amare, 2018; Aiyar et al., 2021a,b). They also offer insights into the gender difference in obesity prevalence across emerging economies (Ameye and Swinnen, 2019), and how this gap is shaped by gender norms across different cultural settings (Costa-Font and Györi, 2020).

These findings carry important implications for public health policy in Pakistan and similar contexts. They reveal that efforts to reduce early marriage — often pursued to improve educational and reproductive outcomes — may also generate long-term health benefits by lowering women’s risk of obesity. With over 40% of women in our sample married before the age of 18 and more than half of adult women classified as overweight or obese, the intersection of these issues is particularly salient. Our results suggest that delaying marriage, especially in urban areas, could serve as a preventive strategy against the rising burden of obesity-related non-communicable diseases. More broadly, the findings highlight the need for holistic, cross-sectoral approaches: female obesity in Pakistan sits at the nexus of gender norms and environmental change. Interventions should therefore address both structural determinants — such as women’s access to education, labour force participation, health knowledge, and family planning—and environmental conditions, including food systems and urban design, alongside culturally sensitive initiatives to delay marriage, such as enforcing legal marriage age and challenging prevailing gender norms.

While this study advances our understanding, several limitations must be acknowledged. First, the analysis is based on cross-sectional DHS data, where obesity is measured only at the time of the survey. As such, we are unable to observe women’s BMI trajectories over the life course and must infer cumulative effects from a single snapshot. Longitudinal data tracking weight changes from before marriage through the post-marriage years, along with repeated measures of potential mediators, would enable a more nuanced analysis of both the timing and mechanisms through which early marriage contributes to obesity.

Second, the PDHS does not collect information on food consumption or energy expenditure, preventing us from examining mechanisms such as the Social Obligation Hypothesis, which posits that individuals may eat more regularly and socially after marriage — a pattern documented in higher-income settings (Averett et al., 2008). Moreover, some of our mediating variables, such as proxies for mental health and indicators of decision-making power, are imperfect and may not fully capture the underlying constructs.

A further limitation concerns the potential endogeneity of age at marriage, which is influenced by natal household characteristics and may be subject to selection bias and omitted variable bias. To address this, we employed a previously validated IV strategy and found that our OLS and IV estimates are consistent. The instrument — community-level norms around marriage age — offers plausibly exogenous variation in individual marriage timing, particularly after controlling for average community socio-economic status. Furthermore, we show that instrumented age at marriage increases the risk of commonly observed adverse fertility-related outcomes associated with child marriage in the existing literature. In addition to offering insight into the mechanisms between early marriage and later obesity, these outcomes are measured closer to the timing of marriage, which provides additional support for the validity of our identification strategy. Nevertheless, residual confounding may persist if unobserved community or household factors, such as culturally determined dietary habits or infrastructural differences, are correlated with both marriage norms and obesity, potentially violating the exclusion restriction.

Future research should prioritise the use of longitudinal or panel data that follow women from adolescence into adulthood to better assess how the timing of marriage shapes long-term health outcomes. In addition, natural experiments, such as policy reforms that alter legal marriage age, could offer valuable quasi-experimental settings for identifying causal effects.

## Supplementary Material

Appendix

## Figures and Tables

**Table 1:** Descriptive statistics

	Pooled			Rural			Urban		
	Mean	SD		Mean	SD		Mean	SD
BMI	25.84	5.69		24.77	5.43		26.91	5.74	
Obese	0.52	0.50		0.43	0.50		0.61	0.49	
Overweight	0.67	0.47		0.59	0.49		0.74	0.44	
Underweight	0.07	0.26		0.10	0.30		0.05	0.22	
Age at marriage	19.11	4.25		18.55	3.91		19.67	4.50	
Child marriage	0.40	0.49		0.45	0.50		0.35	0.48	
Married as a child or adolescent	0.61	0.49		0.67	0.47		0.55	0.50	
Urban	0.50	0.50		0.00	0.00		1.00	0.00	
2017-2018 survey wave	0.53	0.50		0.51	0.50		0.54	0.50	
Age	35.38	8.29		35.12	8.57		35.65	8.00	
Short stature	0.20	0.40		0.20	0.40		0.19	0.40
Average community wealth score	3.10	1.16		2.34	0.90		3.85	0.87	
Observations	6153			3083			3070		

*Notes*. This table displays descriptive statistics for analytical sample of non-pregnant and non-breastfeeding women aged 18-49 with available information on weights and heights from the 2012-2013 and 2017-2018 Pakistan DHS.

**Table 2:** Association between female marital age and obesity

	Pooled				Rural				Urban		
	(1) Obese	(2) Obese	(3) Obese		(4) Obese	(5) Obese	(6) Obese		(7) Obese	(8) Obese	(9) Obese
Age at marriage	0.005*** (0.002)	-0.003* (0.002)	-0.005*** (0.001)		0.005* (0.003)	-0.002 (0.002)	-0.004 (0.002)		0.001 (0.002)	-0.004** (0.002)	-0.006*** (0.002)
Urban		0.153*** (0.018)	0.012 (0.022)								
2017-2018 survey wave		0.122*** (0.017)	0.132*** (0.016)			0.139*** (0.024)	0.134*** (0.023)			0.096*** (0.025)	0.122*** (0.021)
Age		0.011*** (0.001)	0.010*** (0.001)			0.008*** (0.001)	0.008*** (0.001)			0.013*** (0.001)	0.013*** (0.001)
Short stature		0.025 (0.016)	0.032** (0.016)			0.031 (0.023)	0.032 (0.022)			0.009 (0.024)	0.017 (0.024)
Average community wealth score			0.119*** (0.013)				0.149*** (0.025)				0.116*** (0.021)
Constant	0.433*** (0.041)	-0.013 (0.045)	-0.236*** (0.051)		0.348*** (0.058)	0.224*** (0.061)	-0.130 (0.080)		0.608*** (0.043)	-0.206*** (0.066)	-0.498*** (0.074)
Observations	6305	6153	6153		3137	3083	3083		3168	3070	3070
*R* ^2^	0.002	0.161	0.180		0.002	0.182	0.200		0.000	0.151	0.163

*Notes*. This table displays the OLS regression results from Equation 1. The analytical sample consists of non-pregnant and non-breastfeeding women aged 18–49 with available information on weight and height from the 2012–13 and 2017–18 Pakistan DHS. The outcome variable is the probability of being obese (BMI ≥ 25). Columns 1–3, 4–6 and 7–9 show estimates for the pooled, rural and urban samples, respectively. All models include district and survey wave fixed effects. Robust standard errors clustered at the district level are reported in parentheses.

* *p* < 0.10

** *p* < 0.05

*** *p*< 0.01.

**Table 3:** Association between female marital age categories and obesity

	Pooled	Rural	Urban
	(1) Obese	(2) Obese	(3) Obese
Marital age: 17–18	0.004 (0.018)	0.007 (0.026)	-0.005 (0.028)
Marital age: 19	0.008 (0.023)	0.005 (0.030)	0.011 (0.033)
Marital age: 20–22	-0.029 (0.017)	-0.041* (0.024)	-0.014 (0.023)
Marital age: 23+	-0.043** (0.018)	-0.030 (0.031)	-0.059** (0.025)
Controls	**✓**	**✓**	**✓**
Constant	-0.310*** (0.043)	-0.183*** (0.069)	-0.600*** (0.063)
Observations	6153	3083	3070
*R* ^2^	0.180	0.201	0.163

*Notes*. This table displays the OLS regression results from Equation 1. The analytical sample consists of non-pregnant and non-breastfeeding women aged 18–49 with available information on weight and height from the 2012–13 and 2017–18 Pakistan DHS. The outcome variable is the probability of being obese (BMI ≥ 25). The omitted reference category is Marital age: 10–16. All specifications control for urban residence, current age, short stature, average community socio-economic status, and district and survey wave fixed effects. Robust standard errors clustered at the district level are in parentheses.

* *p* < 0.10

** *p* < 0.05

*** *p* < 0.01.

**Table 4:** Association between female marital age and obesity by age groups

	18–24	25–34	35+
	(1) Obese	(2) Obese	(3) Obese
Age at marriage	-0.012 (0.009)	-0.008*** (0.003)	-0.004** (0.002)
Urban	0.005 (0.055)	-0.062* (0.034)	0.037 (0.025)
2017-2018 survey wave	0.150*** (0.049)	0.144*** (0.026)	0.123*** (0.020)
Age	0.023** (0.011)	0.020*** (0.004)	0.002 (0.002)
Short stature	0.056 (0.050)	0.032 (0.031)	0.026 (0.018)
Average community wealth score	0.068** (0.028)	0.143*** (0.018)	0.120*** (0.017)
Constant	-0.077 (0.237)	-0.410*** (0.130)	-0.003 (0.097)
Observations	731	1932	3490
*R^2^*	0.288	0.219	0.179

*Notes*. This table displays OLS regression results across age groups. The analytical sample consists of non-pregnant and non-breastfeeding women aged 18–49 with available weight and height data from the 2012–13 and 2017–18 Pakistan DHS. The outcome is the probability of being obese (BMI ≥ 25). Columns 1–3 show estimates for women currently aged 18–24, 25–34, and 35+ years, respectively. All models include district fixed effects. Robust standard errors clustered at the district level are shown in parentheses.

* *p* < 0.10

** *p* < 0.05

*** *p* < 0.01.

**Table 5:** Association between female marital age and obesity by birth cohorts

	1962–1974	1975–1984	1985–2000
	(1) Obese	(2) Obese	(3) Obese
Age at marriage	-0.002 (0.003)	-0.005** (0.002)	-0.010*** (0.004)
Urban	0.045 (0.038)	0.006 (0.030)	-0.028 (0.031)
2017-2018 survey wave	0.132*** (0.027)	0.092*** (0.031)	0.151*** (0.031)
Age	0.003 (0.004)	0.013*** (0.004)	0.021*** (0.003)
Short stature	0.063** (0.025)	0.012 (0.026)	0.033 (0.033)
Average community wealth score	0.126*** (0.023)	0.111*** (0.020)	0.116*** (0.018)
Constant	-0.099 (0.165)	-0.020 (0.127)	-0.451*** (0.101)
Observations	2049	2252	1852
*R* ^2^	0.223	0.186	0.241

*Notes*. This table displays the OLS regression results from Equation 1 by birth cohort groups. The analytical sample consists of non-pregnant and non-breastfeeding women aged 18–49 with available information on weight and height from the 2012–13 and 2017–18 Pakistan DHS. The outcome variable is the probability of being obese (BMI ≥ 25). Columns 1–3 show estimates for women born in 1962–1974, 1975–1984, and 1985–2000, respectively. All specifications include district fixed effects. Robust standard errors clustered at the district level are in parentheses.

* *p* < 0.10

** *p* < 0.05

*** *p* < 0.01.

**Table 6:** Association between marital age and obesity by household wealth

	(1) Poorest	(2) Poorer	(3) Middle	(4) Richer	(5) Richest
	Obese	Obese	Obese	Obese	Obese
Age at marriage	-0.009* (0.005)	-0.001 (0.004)	-0.003 (0.004)	-0.009** (0.004)	-0.008*** (0.002)
Urban	0.019 (0.069)	0.015 (0.055)	-0.011 (0.045)	0.010 (0.051)	0.080** (0.036)
2017-2018 survey wave	0.057 (0.048)	0.151*** (0.039)	0.181*** (0.037)	0.096*** (0.035)	0.147*** (0.022)
Age	0.007*** (0.002)	0.007*** (0.002)	0.011*** (0.002)	0.010*** (0.002)	0.016*** (0.001)
Short stature	0.053 (0.035)	0.013 (0.038)	0.009 (0.039)	0.052 (0.036)	0.060* (0.036)
Average community wealth	-0.007 (0.054)	0.066* (0.035)	0.097*** (0.034)	0.070* (0.036)	0.036 (0.031)
Constant	0.535*** (0.151)	-0.204* (0.121)	-0.223* (0.133)	0.208 (0.142)	-0.217 (0.151)
Observations	934	1216	1184	1268	1551
*R* ^2^	0.291	0.224	0.229	0.158	0.213

*Notes*. This table displays the OLS regression results from Equation 1 by household wealth quintile. The analytical sample consists of non-pregnant and non-breastfeeding women aged 18–49 with available weight and height data from the 2012–13 and 2017–18 Pakistan DHS. The outcome variable is the probability of being obese (BMI ≥ 25). Columns 1–5 show estimates for the poorest, poorer, middle, richer, and richest wealth quintiles, respectively. All models include district fixed effects. Robust standard errors clustered at the district level are shown in parentheses.

* *p* < 0.10

** *p* < 0.05

*** *p* < 0.01.

**Table 7:** Effect of marital age on obesity - IV regression results

	Pooled		Rural		Urban
					
Age at marriage	-0.002 (0.004)		0.004 (0.007)		-0.007* (0.004)
Urban	0.006 (0.022)				
2017-2018 survey wave	0.120***		0.108***		0.122***
	(0.016)		(0.024)		(0.021)
Age	0.012***		0.009***		0.014***
	(0.001)		(0.001)		(0.001)
Short stature	0.035**		0.044*		0.008
	(0.017)		(0.024)		(0.025)
Average community wealth score	0.111*** (0.014)		0.139*** (0.026)		0.107*** (0.021)
Observations	5463		2704		2759
*R* ^2^	0.088		0.059		0.071
Kleibergen-Paap rk Wald F statistic	412.767		157.625		198.338

*Notes:* This table displays the second stage IV regression results. The sample includes urban, non-pregnant, non-breastfeeding women aged 18–49 from the 2012–13 and 2017–18 Pakistan DHS. The dependent variable is a binary indicator for obesity (BMI ≥ 25). The instrument is the average age of marriage per DHS cluster, five years prior to a given woman’s year of marriage. All specifications control for district fixed effects. Robust standard errors clustered on district are presented in parentheses.

* *p* < 0.10

** *p* < 0.05

*** *p* < 0.01.

**Table 8:** Effects of female marital age on human capital - IV regression results for the urban sample

	(1)	(2)	(3)	(4)	(5)
	Non-poor	Woman’s years of schooling	Literate	High health knowledge	Employed
Age at marriage	-0.001 (0.004)	0.482*** (0.058)	0.026*** (0.004)	0.018*** (0.004)	0.009** (0.004)
Controls	✓	✓	✓	✓	✓
Mean Dep. Var.	0.134	6.247	0.588	0.616	0.189
Observations	2765	2765	2765	2765	2764
*R* ^2^	0.117	0.250	0.148	0.101	0.018

This table displays the IV regression results for potential mechanisms explaining the relationship between marital age and obesity. The sample includes urban, non-pregnant, non-breastfeeding women aged 18–49 from the 2012–13 and 2017–18 Pakistan DHS. Marital age is instrumented with the average marital age per DHS cluster five years prior to a given woman’s year of marriage. Robust standard errors clustered at the district level are shown in parentheses. All specifications include district fixed effects.

* *p* < .10

** *p* < .05

*** *p* < .01

**Table 9:** Effects of female marital age on fertility - IV regression results for the urban sample

	(1) Have children	(2) Age at first birth	(3) Teen pregnancy	(4) Number of children	(5) Number of short birth intervals
Age at marriage	-0.019***	0.879***	-0.039***	-0.212***	-0.016***
	(0.002)	(0.026)	(0.004)	(0.016)	(0.006)
Controls	✓	✓	✓	✓	✓
Mean Dep. Var.	0.855	21.536	0.172	3.286	0.166
Observations	2765	2365	2365	2765	2077
*R* ^2^	0.241	0.771	0.231	0.509	0.024

This table displays the IV regression results for potential mechanisms explaining the relationship between marital age and obesity. The sample includes urban, non-pregnant, non-breastfeeding women aged 18–49 from the 2012–13 and 2017–18 Pakistan DHS. Marital age is instrumented with the average marital age per DHS cluster five years prior to a given woman’s year of marriage. Robust standard errors clustered at the district level are shown in parentheses. All specifications include district fixed effects.

* *p* < .10

** *p* < .05

*** *p* < .01

**Table 10:** Effects of female marital age on female decision making - IV regression results for the urban sample

	(1) Restricted mobility	(2) No decision-making power	(3) Experience of domestic violence	(4) Age difference with husband
Age at marriage	-0.002	-0.003	-0.002	-0.336***
	(0.006)	(0.005)	(0.005)	(0.077)
Controls	✓	✓	✓	✓
Mean Dep. Var.	0.225	0.310	0.323	5.567
Observations	2765	2765	2190	2622
*R* ^2^	0.022	0.037	0.015	0.072

This table displays the IV regression results for potential mechanisms explaining the relationship between marital age and obesity. The sample includes urban, non-pregnant, non-breastfeeding women aged 18–49 from the 2012–13 and 2017–18 Pakistan DHS. Marital age is instrumented with the average marital age per DHS cluster five years prior to a given woman’s year of marriage. Robust standard errors clustered at the district level are shown in parentheses. All specifications include district fixed effects.

* *p* < .10

** *p* < .05

*** *p* < .01

## Footnotes

1 Other forms of selection have been proposed in the literature on marital status and BMI in higher-income contexts. For example, women with a high BMI may be less likely to marry or may marry later (Averett et al., 2008). Additionally, Tang et al. (2024) suggest that earlier marriage in China may be associated with different spousal preferences — such as physical appearance at younger ages and financial status or career achievements at older ages. However, selection into age at marriage based on pre-marital BMI is unlikely to be a concern in our data from Pakistan, where 75% of marriages in the sample took place between 1979 and 2006. In Pakistan, 99.7% of adults over the age of 65 have ever been married, and marriages are traditionally arranged. The bride and groom often do not meet before the wedding, and the timing and choice of marriage partner are typically made by the families. Traditionally, girls are married soon after reaching physical maturity (Agha, 2016; Habiba et al., 2016; Veen et al., 2018; Jensen and Thornton, 2003; Rasul et al., 2022). As most couples reside with the groom’s family, the bride is often selected based on her perceived ability to contribute to the household (Agha, 2016; Habiba et al., 2016; Veen et al., 2018; Rasul et al., 2022).

2 We do not find a statistically significant relationship between obesity and a binary variable indicating whether the respondent was married before or after age 18.

3 The results are qualitatively similar if restricting the sample to women who did not migrate since birth.
