# Supplementary material for: Women’s Age at Marriage and Obesity in Pakistan
Source: Econ Hum Biol. Author manuscript; Available in PMC 2025 Oct 7. (PMC7618209; doi:10.1016/j.ehb.2025.101525)
Supplement: Appendix [file EMS209378-supplement-Appendix.pdf]

7. Appendix

Table A1: Association between female marital age and obesity controlling for district-trends and DHS cluster fixed effects

	Pooled		Rural		Urban	
	(1)	(2)	(3)	(4)	(5)	(6)
	Obese	Obese	Obese	Obese	Obese	Obese
Age at marriage	-0.004***	-0.005***	-0.004	-0.004*	-0.005***	-0.005**
	(0.001)	(0.002)	(0.002)	(0.002)	(0.002)	(0.002)
District x survey year	✓		✓		✓	
DHS cluster fixed effect		✓		✓		✓
Constant	-0.150***	0.253***	-0.164**	0.258***	-0.189**	0.236***
	(0.051)	(0.043)	(0.071)	(0.053)	(0.079)	(0.062)
Observations	6147	6137	3079	3079	3066	3058
R^2	0.211	0.325	0.248	0.326	0.200	0.286

Notes. This table displays the OLS regression results from Equation 1 for the pooled, rural and urban samples. The analytical sample consists of non-pregnant and non-breastfeeding women aged 18-49 with available information on weights and heights from the 2012-2013 and 2017-2018 Pakistan DHS. The outcome variable is the probability of being obese (BMI $\geq$ 25). All specifications control for survey wave, urban residence, current age and short stature. columns 1, 3 and 5 additionally control for district level trends and average community wealth quintile. Columns 2, 4 and 6 additionally control for DHS cluster fixed effects. Robust standard errors clustered on district are presented in parentheses. * $p < .10$, ** $p < .05$, *** $p < .01$

Table A2: Association between female marital age and risk of overweight

	Pooled (Overweight)			Rural (Overweight)			Urban (Overweight)		
	(1)	(2)	(3)	(4)	(5)	(6)	(7)	(8)	(9)
Age at marriage	0.005***	-0.002	-0.004**	0.002	-0.005*	-0.006**	0.003	-0.002	-0.003
	(0.002)	(0.002)	(0.001)	(0.003)	(0.002)	(0.002)	(0.002)	(0.002)	(0.002)
Urban		0.131***	0.011						
		(0.018)	(0.023)						
2017–2018 survey wave		0.106***	0.114***		0.122***	0.118***		0.080***	0.097***
		(0.018)	(0.017)		(0.026)	(0.025)		(0.019)	(0.017)
Age		0.011***	0.010***		0.010***	0.009***		0.011***	0.011***
		(0.001)	(0.001)		(0.001)	(0.001)		(0.001)	(0.001)
Short stature		0.039**	0.045***		0.046*	0.047*		0.034	0.039*
		(0.017)	(0.017)		(0.024)	(0.024)		(0.022)	(0.023)
Average community wealth score			0.102***			0.128***			0.078***
			(0.014)			(0.025)			(0.020)
Constant	0.577***	0.178***	-0.012	0.553***	0.472***	0.168*	0.688***	-0.074	-0.271***
	(0.038)	(0.046)	(0.054)	(0.057)	(0.068)	(0.088)	(0.039)	(0.055)	(0.074)
Observations	6305	6153	6153	3137	3083	3083	3168	3070	3070
R^2	0.002	0.163	0.178	0.000	0.201	0.215	0.001	0.143	0.150

Notes. This table displays the OLS regression results from Equation 1. The analytical sample consists of non-pregnant and non-breastfeeding women aged 18–49 with available information on weight and height from the 2012–13 and 2017–18 Pakistan DHS. The outcome variable is being overweight (BMI ≥ 23). Columns 1–3, 4–6, and 7–9 show estimates for the pooled, rural, and urban samples, respectively. All specifications include district fixed effects. Robust standard errors clustered at the district level are in parentheses. * $p < 0.10$, ** $p < 0.05$, *** $p < 0.01$.

Table A3: Association between female marital age and BMI

	Pooled			Rural			Urban		
	(1)	(2)	(3)	(4)	(5)	(6)	(7)	(8)	(9)
	BMI	BMI	BMI	BMI	BMI	BMI	BMI	BMI	BMI
Age at marriage	0.061***	-0.032**	-0.060***	0.045	-0.045	-0.069***	0.015	-0.041*	-0.059***
	(0.021)	(0.016)	(0.016)	(0.032)	(0.028)	(0.026)	(0.023)	(0.022)	(0.022)
Urban		1.913***	0.067						
		(0.237)	(0.243)						
2017–2018 survey wave		1.416***	1.548***		1.715***	1.649***		1.040***	1.313***
		(0.194)	(0.180)		(0.271)	(0.254)		(0.250)	(0.222)
Age		0.137***	0.131***		0.107***	0.104***		0.167***	0.163***
		(0.009)	(0.009)		(0.010)	(0.009)		(0.013)	(0.013)
Short stature		0.637***	0.725***		0.800***	0.824***		0.431	0.510
		(0.211)	(0.211)		(0.273)	(0.265)		(0.311)	(0.315)
Average community wealth score			1.560***			2.068***			1.212***
			(0.155)			(0.277)			(0.229)
Constant	24.677***	19.356***	16.435***	23.933***	21.262***	16.358***	26.610***	19.075***	16.025***
	(0.449)	(0.506)	(0.605)	(0.627)	(0.722)	(0.982)	(0.542)	(0.655)	(0.867)
Observations	6148	6148	6148	3080	3080	3080	3068	3068	3068
R^2	0.002	0.175	0.199	0.001	0.204	0.233	0.000	0.161	0.171

Notes. This table displays the OLS regression results from Equation 1. The analytical sample consists of non-pregnant and non-breastfeeding women aged 18–49 with weight and height data from the 2012–13 and 2017–18 Pakistan DHS. The outcome variable is BMI. Columns 1–3, 4–6, and 7–9 show results for pooled, rural, and urban samples, respectively. District fixed effects are included. Standard errors are clustered at the district level. * $p < 0.10$, ** $p < 0.05$, *** $p < 0.01$.

Table A4: Association between female marital age and risk of underweight

	Pooled (Underweight)			Rural (Underweight)			Urban (Underweight)		
	(1)	(2)	(3)	(4)	(5)	(6)	(7)	(8)	(9)
Age at marriage	-0.002*	0.000	0.001	-0.000	0.001	0.001	-0.001*	-0.000	0.000
	(0.001)	(0.001)	(0.001)	(0.002)	(0.001)	(0.001)	(0.001)	(0.001)	(0.001)
Urban		-0.040***	-0.004						
		(0.010)	(0.008)						
2017–2018 survey wave		-0.031***	-0.034***		-0.041***	-0.040***		-0.018**	-0.021**
		(0.008)	(0.008)		(0.012)	(0.012)		(0.008)	(0.008)
Age		-0.003***	-0.003***		-0.003***	-0.003***		-0.003***	-0.003***
		(0.000)	(0.000)		(0.001)	(0.001)		(0.001)	(0.001)
Short stature		-0.018*	-0.020**		-0.028*	-0.029*		-0.011	-0.012
		(0.010)	(0.010)		(0.015)	(0.015)		(0.010)	(0.011)
Avg. community wealth score			-0.031***			-0.032**			-0.013*
			(0.006)			(0.012)			(0.008)
Constant	0.104***	0.233***	0.291***	0.105***	0.190***	0.264***	0.073***	0.247***	0.281***
	(0.018)	(0.022)	(0.028)	(0.030)	(0.037)	(0.050)	(0.015)	(0.027)	(0.034)
Observations	6305	6153	6153	3137	3083	3083	3168	3070	3070
R^2	0.001	0.084	0.089	0.000	0.121	0.123	0.001	0.072	0.073

Notes. This table displays the OLS regression results from Equation 1. The analytical sample consists of non-pregnant and non-breastfeeding women aged 18–49 with height and weight data from the 2012–2013 and 2017–2018 Pakistan DHS. The outcome is underweight (BMI < 18.5). Columns 1–3, 4–6, and 7–9 show pooled, rural, and urban estimates, respectively. All models include district fixed effects. Robust standard errors clustered at the district level in parentheses. * $p < 0.10$, ** $p < 0.05$, *** $p < 0.01$.

Table A5: Association between female marital age and obesity: Logit regression results

	Pooled			Rural			Urban		
	(1)	(2)	(3)	(4)	(5)	(6)	(7)	(8)	(9)
Odds ratios	Obese	Obese	Obese	Obese	Obese	Obese	Obese	Obese	Obese
Age at marriage	1.021***	0.987*	0.978***	1.021*	0.991	0.983	1.002	0.979**	0.970***
	(0.008)	(0.007)	(0.007)	(0.012)	(0.011)	(0.011)	(0.009)	(0.009)	(0.009)
Urban		2.043***	1.057						
		(0.176)	(0.113)						
2017–2018 survey wave		1.787***	1.897***		1.960***	1.923***		1.612***	1.854***
		(0.145)	(0.145)		(0.225)	(0.216)		(0.195)	(0.188)
Age		1.052***	1.051***		1.042***	1.042***		1.065***	1.064***
		(0.004)	(0.004)		(0.005)	(0.005)		(0.006)	(0.006)
Short stature		1.128	1.167**		1.167	1.180		1.040	1.084
		(0.084)	(0.089)		(0.130)	(0.131)		(0.119)	(0.126)
Avg. community wealth score			1.761***			2.057***			1.774***
			(0.111)			(0.258)			(0.181)
Constant	0.762*	0.089***	0.029***	0.542***	0.233***	0.040***	1.549**	0.036***	0.008***
	(0.125)	(0.020)	(0.008)	(0.128)	(0.067)	(0.016)	(0.282)	(0.012)	(0.003)
Observations	6305	6129	6129	3137	3028	3028	3168	3043	3043
Log pseudolikelihood	-4351.826	-3717.136	-3652.238	-2152.099	-1795.937	-1764.168	-2107.192	-1807.242	-1785.190
Pseudo R^2	0.001	0.124	0.139	0.001	0.134	0.150	0.000	0.116	0.127

Notes. This table displays logit regression results based on Equation 1. The analytical sample consists of non-pregnant and non-breastfeeding women aged 18–49 with height and weight data from the 2012–2013 and 2017–2018 Pakistan DHS. The dependent variable is obesity (BMI $\geq$ 25). Columns 1–3, 4–6, and 7–9 show pooled, rural, and urban samples, respectively. Odds ratios are reported. District fixed effects are included. Robust standard errors clustered at the district level are in parentheses. * $p < 0.10$, ** $p < 0.05$, *** $p < 0.01$.
